# 

**Published:** 1890-12

**Authors:** 


The Papers and Discussions will be continued in the January Number. Editor.

### DIRECTORY OF DENTAL SOCIETIES.

*American Dental Association*—Meets on the first Tuesday of August, 1890, at Excelsior Springs, Mo. Pres., M. W. Foster, Baltimore, Md.; Rec. Sec., Geo. H. Cushing, Chicago.

*Southern Dental Association*—Meets in Atlanta, Ga., on the third Tuesday in July, 1890. Prest., J. C. Story, Dallas, Texas; Secretary, M. C. Marshall, Little Rock, Ark.

### STATE DENTAL SOCIETIES.

*Alabama Dental Association*—Meets annually. Next meeting at Birmingham, on the second Tuesday of April, 1890. Pres. J. C. Wilkerson, Selma; Sec. R. Y. Jones, Florence.

*California State Dental Association*—Third Tuesday in July, 1890. Pres., T. N. Iglehart; Rec. Sec., W. A. Knowles, San Francisco; Cor. Sec., J. J. Biege. Next Meeting in San Francisco.

*Colorado State Dental Society*—Meets first Tuesday in June, 1890. Pres., J. W. Grannis; Sec., H. P. Kellog, Denver. Next meeting will be held in Denver.

*Connecticut State Dental Society*—Pres. E. A. Stebbins, Shelburne Falls; Rec. Sec. Geo. A. Maxfield, Holyoke. Annual meeting Oct. 27th and 28th, 1889, at Hartford.

*Dental Society of the State of Maryland and District of Columbia*—Meets annually. Next meeting at Washington City, on the second Tuesday in October, 1889. Pres. B. F. Coy, of Baltimore: Sec. M. W. Foster, M. D.

*Georgia State Dental Society*—Meets second Tuesday in May, 1890, at Tybee; Pres. S. A. White: Rec. Sec. H. H. Johnson; Cor. Sec. L. D. Carpenter, Atlanta.

*Illinois State Dental Society*—Meets at Springfield, second Tuesday in May, 1890. Pres., T. W. Pritchett, Chicago; Sec., G. Newkirk, Chicago.

*Indiana State Dental Society*—Meets next in Indianapolis on the last Tuesday of June, 1890. Pres., T. A. Goodwin, Warsaw, Ind.; Sec. Robt. W. Van Valzah, Terre Haute.

*Iowa State Dental Society*—Meets annually. Next meeting in Dubuque, on the first Tuesday in May, 1890. Pres. F. M. Shriver, Glenwood; Rec. Sec. G. W. Miller, Des Moines.

*Kentucky State Dental Association*—Meets annually, first Tuesday in June, 1890. Next meeting in Louisville. Pres., J. W. Wallace, Louisville; Sec., J. H. Baldwin, Louisville

*Kansas State Dental Association*—Pres. W. M. Shirley, Hiawatha; Sec. C. B. Gunn, Leavenworth. Next meeting will be held at Topeka, April 30th, 1890.

*Maine Dental Society*—Meets in August, at Thomaston. Pres. E. J. Robert, Augusta, Sec. C. E. Hussey, Beddeford.

*Maryland State Dental Association*—Meets annually in December. Pres. J. Emory Scott; Rec. Sec. W. W. Dunbracco; Cor. Sec.

*Massachusetts Dental Society*—Meets Semi-Annually in Boston, June 5th, 6th and 7th, 1890. Pres. G. A. Gerry, Boston; Rec. Sec. E. O. Kinsman, Boston.

*Michigan State Dental Association*—Meets annually. Next meeting at Jackson, Tuesday, June 7th, 1890. Pres. C. S. Case, Jackson; Sec. Wm. Cleland, Detroit; Treas. H. K. Lathrop, Detroit.

*Mississippi State Dental Association*—Meets at Biloxi, 1890, during Camp Meeting. Pres., J. D. Miller, Vicksburg; Sec., A. H. Hilzim, Jackson.

*Minnesota State Society*—Pres. L. E. Weeks, Minneapolis; Sec. D. F. Mac Graw, Mankato. Meets at Deluth, 3rd Tuesday in July, for four days.

*Missouri Dental Association*—Meets at Pertel Springs, first Tuesday after July 4th, 1890. Pres., H. Fisher, St. Louis; Sec., John G. Harper, St. Louis.

*Nebraska State Dental Society*—Meets annually. Next meeting third Tuesday in May 1890, at Beatrice; Pres. J. J. Willey, Wahoo; Sec. C. R. Tefft, Lincoln.

*New Jersey State Dental Society*—Meets annually. Next meeting at Asbury Park, on the third Wednesday in July, 1890. Pres. S. C. G. Watkins, Mount Clair; Sec. C. A. Meeker, Newark.

*New Hampshire Dental Society*—Meets in Concord on the third Tuesday of June, 1890. Pres., J. C. Duncan, East Jaffery; Sec., E. B. Davis, Concord.

*North Carolina State Dental Society*—Meets in Wilmington, on the fourth Wednesday in June, 1890. Pres. S. P. Hilliard; Sec., H. C. Hearing, Concord.

*North Dakota Dental Society*—Meets ——, 1890. Pres., I. J. Hill, Fargo; Sec., J. W. Cloes, Jamestown.

*Ohio State Dental Society*—Meets annually. Next meeting at Columbus, last Tuesday of October, 1890. Pres. J. N. Sedgwick, Granville; Sec. J. R. Callahan, Cincinnati.

*Pennsylvania State Dental Society*—Meets second Tuesday of September, 1889, at Philadelphia; Pres. W. H. Roop Philadelphia; Rec. and Cor. Sec. Th. F. Chupein, Philadelphia.

*Rhode Island Dental Association*—Pres . H. W. Gillett; Sec., W. H. Carpenter. Meets quarterly, first Tuesdayin July, October, January, and April. Annual Meeting first Tuesday in January, at Providence.

*South Carolina State Dental Association*—Meets annually. Pres. L. S. Wolfe, Orangburg; Cor. Sec. E. C. Ridgell, Batesburg; Rec. Sec. R. A. Smith, Charleston. Next meeting at Greenville, third Tuesday in July, 1890.

*Texas State Dental Association*—Meets in Buton, first Tuesday in May, 1890. Pres., M. S. Read, Corsicana; Sec., C. B. Lewis, Dallas.

*Tennessee Dental Association*—Meets Annually Next meeting at Jackson, first Tuesday in July 1890. Pres. A. Gordon White, Springfield; Rec. Sec. J. D. Hanston, Lewisburg; Cor. Sec. Gordon White, Nashville.

*The Dental Society of the State of New York*—Meets annually on the second Wednesday in May. Next session at Albany, May 8, 1890. Pres. J. Edw. Line, Rochester; Rec. Sec., F. T. Van Wert, Brooklyn; Cor. Sec., G. L. Curtis, Syracuse.

*Vermont State Dental Society*—Meets annually. Next meeting at Bellow's Falls, March 19, 1890. Pres. W. H. Spence, Poultney; Sec. Thos. Maund, Rutland.

*Virginia State Dental Association*—Meets in Charlottsville, Tuesday, August 19, 1890. Pres. J. Hall Moore; Sec. L. M. Cowarden, Richmond.

*Wisconsin State Dental Society*—Meets annually. Next meeting at Milwaukee, July 20, 1890 Pres. B. G. Marklein, Milwaukee; Sec. C. A. Southwell, Appleton, Wis.

### LOCAL SOCIETIES.

*American Academy of Dental Science*—Meets annually in Boston, Mass., on the first Wednesday in October, 1890. Pres. J. H. Batchelder; Rec. Sec. E. E. Hopkins; Cor. Sec. E. B. Hitchcock, Boston.

*Brooklyn Dental Society and Second District Society United*—Meets quarterly—Pres. T. H. Holly; Rec. Sec. A. N. Russell, Brooklyn.

*Central Dental Association of Northern New Jersey*—Meets bi-monthly; and annually in February. Pres. Oscar Adelburg, Elizabeth; Sec. Geo. Emory Adams, South Orange.

*Central Illinois Dental Society*—Meets annually. Next meeting at Lincoln, Oct. Oct. 9 and 10, 1889; Pres. J. M. Fishburn, El Paso; Sec. W. A. Johnston, Peoria.

*Chicago Dental Society*—Meets monthly. Pres. P. J. Kester; Sec. A. E. Baldwin.

*Chicago Dental Club*—Meets on the fourth Monday of each month. Pres. A. B. Freeman; Sec. C. Stoddard Smith.

*Cleveland Dental Society.*—Meets the first Monday of each month. Pres. S. B. Dewey; Sec. Martha J. Robinson.

*Connecticut Valley Dental Society*—Meets 27 and 28 of Oct. 1889, at Savin Rock Conn. Pres. C. T. Stockwell, Springfield, Mass. Sec. A. M. Ross, Chicopee.

*Detroit Dental Society*—Meets monthly, Pres. E. C. Moore, Sec. Wm. Cleland.

*Eighth District Dental Society, N. Y.*—Meets semi-annually. Next meeting in Syracuse, Oct. 24-26, 1889. Pres. T. E. Howard; Sec. S. A. Freeman, Buffalo.

*East Tennessee Dental Association*—Meets annually. Next meeting will be held at Chattanooga, Tenn., on the first Tuesday of Aug., 1890. Pres. R. A. B. Noyers, Sec. S. N. Prothro.

*Mad River Dental Society*—Meets annually. Next meeting at Dayton, Tuesday, May 19, 1889. Pres. C. Bradley, Dayton; Sec. and Treas. L. C. Adams, Dayton,

*Mississippi Valley Dental Society*—Meets annually at Cincinnati, on first Wednesday in March, 1890. Pres. ................................ Sec. .............. Cincinnati.

*New England Dental Society*—Meets annually. Time and place of next meeting in Boston, November, 1890. Pres., C. W. Clement, Manchester, N. H.; Sec. E. O. Kinsman, Cambridge, Mass.

*Northern Illinois Dental Society*—Meets annually. Next meeting at Freeport, October 15th, 1890. Pres. G. W. Dennis, La Salle. Sec. T. W. Beckwith.

*Northern Ohio Dental Association*—Meets annually. Next meeting at Canton, O. on the second Tuesday in May, 1890. Pres. John Stephan, Cleveland, O.; Rec. Sec. F. F. Doud; Cor. Sec. S. B. Dewey, Cleveland, O.

*Northwestern Dental Association* (embraces portions of Dakota, Minnesota and Manitoba) meets annually. Next meeting at Detroit, Dak. on the fourth Tuesday in July, 1890. Pres. J. W. Cloes, Jamestown, Dak. Sec. S. J. Hill, Fargo, Dak.

*Odontological Society of Pennsylvania*—Meets on the first Saturday evening of each month, at 8 o'clock p. m., at 13th and Arch sts; Pres. Jas. Truman, Rec. Sec. A W. Deane.

*Odontological Society of New York City*—Meets the third Tuesday of each month. Pres. Wm. Jarvie; Rec. Sec. E. T. Payne.

*Odontological Society of Cincinnati*—Meets monthly. Pres. C. M, Wright; Sec W. M. Williams.

*Ohio Valley Dental Society.*—Meets quarterly. Next meeting first Monday in July, 1890, at Stuebenville, O. Pres. N. H. Harrison, Cadiz; Sec'y F. S. Maxwell, Stuebenville, O.

*Pennsylvania Association of Dental Surgeons*—Meets second Tuesday of each month; Pres. H. E. Roberts; Cor. Sec. and Reporter, W. N. Truman.

*Pittsburg Dental Society*—Meets the last Tuesday Evening of each month. Pres. J. B. Williams, Homestead; Sec. N. L. Reinecke.

*Fifth District Dental Society of N. Y.*—Meets semi-annually. Next meeting in Syracuse, October, 24-26. Pres. R. F. Jones, Utica; Sec. T. B. Mason, Phoenix.

*San Francisco Dental Society*—Meets the second Monday evening of each month. Pres. W. A. Knowles; Sec. Chas. Morffew; Treas. J. J. Birge.

*Sixth District Dental Society of N. Y.*—Meets semi-annually. Next meeting at Syracuse, Oct. 24-26, 1889. Pres. E. D. Downs, Owego; Sec. Myron D. Jewell, Richfield Springs.

*St. Louis Dental Society*—Meets first and third Tuesday in each month. Pres. A. J. Prosser; Cor. Sec. Wm. Conrad; Rec. Sec. Jessie E. Grosheider.

*Washington Dental Society*—Meets second Tuesday of each month. Pres. R. Finley Hunt; Rec. Sec. H. M. Schooly.

*First District Dental Society of the State of New York*—Meets annually. Pres. A. I. Northrop; Sec. J. E. Dexter, New York.

*Washington City Dental Society*—Pres. M. B. Noble; Sec. Wm. Donally. Meets monthly.

*The Western Illinois District Dental Association*—Meets at Kewanee, October 16, Pres. F. Christianer, Abington; Sec. R. W. Bailey, Macomb.

---

### EXCHANGES

St. Louis, Medical and Surgical Journal.

The Pacific Medical and Surgical Journal, San Francisco.

Cincinnati Lancet and Clinic.

Dental Cosmos, Philadelphia.

Chicago Medical Examiner.

American Journal of Dental Science, Baltimore.

Archives of Dentistry.

L'art Dentaire, Paris.

Le Progres Dentare, Paris.

Deutsche Monatsschrift fuer Zahnheilkunde Leipzig.—Arthur Felix.

Ohio State Journal of Dental Science, Xenia, Ohio

The International Dental Journal, Philadelphia, Pa.

The Dental Record, London.

The Journal of the British Dental Association, London.

The Southern Dental Journal.

Items of Interest, Philadelphia.

The Dental Practitioner.—The Cincinnati "Medical" News.

The British Journal of Dental Science, London, Eng.

The Dental Advertiser.

The Messenger of Dentistry, Russian.

The Medical World.

The American Pharmacist.

The Nashville Journal of Medicine and Surgery.

Medical and Surgical Reporter, Philadelphia, Pa.

Dental Headlight.

Cincinnati Medical and Dental Journal.

The Dental Review.

The Western Dental Journal.

ESTABLISHED 1845.

### Ohio College of Dental Surgery.

Department of Dentistry—University of Cincinnati.

### SESSION 1888-89.

### FACULTY.

J. S. CASSIDY, M.D., D D.S., Professor of Chemistry and Materia Medica.
H. A. SMITH, D.D.S., DEAN OF THE FACULTY, Professor of Operative Dentistry and Dental Pathology.
C. M. WRIGHT, D.D.S., Professor of Physiology and General Pathology.
WM. KNIGHT, M.D., D.D.S., Professor of Anatomy and Oral Surgery.
GRANT MOLLYNEAUX, D.D.S., Professor of Mechanical Dentistry and Metallurgy.

### DEMONSTRATORS.

A. O. ROSS, D.D.S.,
F. L. CARY, D.D.S., } Demonstrators of Operative Dentistry.
G. S. JUNKERMAN, M.D., D.D.S., Demonstrator of Analytical Chemistry.
H. C. MATLACK, D.D.S., Demonstrator of Anatomy.
G. W. GANDEE, D.D.S., Demonstrator of Mechanical Dentistry.

### The Forty-Fourth Annual Session

BEGINS TUESDAY, OCT. 1st, 1889.

### FEES.

| | |
|---|---|
| Marticulation Fee (but once) | $ 5.00 |
| Professors' Tickets | 75.00 |
| Dissecting Ticket, including Material | 10.00 |
| Analytical Chemistry | 10.00 |
| Diploma Fee | 25.00 |
| Spring Course Ticket | 20.00 |

For further information and announcements, address

H. A. SMITH, Dean, 128 Garfield Place, Cincinnati, Ohio.

ESTABLISHED 1845.

### Ohio College of Dental Surgery.

Department of Dentistry—University of Cincinnati.

### SESSION 1888-89.

### FACULTY.

J. S. CASSIDY, M.D., D D.S., Professor of Chemistry and Materia Medica.
H. A. SMITH, D.D.S., DEAN OF THE FACULTY, Professor of Operative Dentistry and Dental Pathology.
C. M. WRIGHT, D.D.S., Professor of Physiology and General Pathology.
WM. KNIGHT, M.D., D.D.S., Professor of Anatomy and Oral Surgery.
GRANT MOLLYNEAUX, D.D.S., Professor of Mechanical Dentistry and Metallurgy.

### DEMONSTRATORS.

A. O. ROSS, D.D.S.,
F. L. CARY, D.D.S., } Demonstrators of Operative Dentistry.
G. S. JUNKERMAN, M.D., D.D.S., Demonstrator of Analytical Chemistry.
H. C. MATLACK, D.D.S., Demonstrator of Anatomy.
G. W. GANDEE, D.D.S., Demonstrator of Mechanical Dentistry.

### The Forty-Fourth Annual Session

BEGINS TUESDAY, OCT. 1st, 1889.

### FEES.

| | |
|---|---|
| Marticulation Fee (but once) | $ 5.00 |
| Professors' Tickets | 75.00 |
| Dissecting Ticket, including Material | 10.00 |
| Analytical Chemistry | 10.00 |
| Diploma Fee | 25.00 |
| Spring Course Ticket | 20.00 |

For further information and announcements, address

H. A. SMITH, Dean, 128 Garfield Place, Cincinnati, Ohio.

ESTABLISHED 1845.

### Ohio College of Dental Surgery.

Department of Dentistry—University of Cincinnati.

### SESSION 1888-89.

### FACULTY.

J. S. CASSIDY, M.D., D D.S., Professor of Chemistry and Materia Medica.
H. A. SMITH, D.D.S., DEAN OF THE FACULTY, Professor of Operative Dentistry and Dental Pathology.
C. M. WRIGHT, D.D.S., Professor of Physiology and General Pathology.
WM. KNIGHT, M.D., D.D.S., Professor of Anatomy and Oral Surgery.
GRANT MOLLYNEAUX, D.D.S., Professor of Mechanical Dentistry and Metallurgy.

### DEMONSTRATORS.

A. O. ROSS, D.D.S., F. L. CARY, D.D.S., } Demonstrators of Operative Dentistry.
G. S. JUNKERMAN, M.D., D.D.S., Demonstrator of Analytical Chemistry.
H. C. MATLACK, D.D.S., Demonstrator of Anatomy.
G. W. GANDEE, D.D.S., Demonstrator of Mechanical Dentistry.

### The Forty-Fourth Annual Session

BEGINS TUESDAY, OCT. 1st, 1889.

### FEES.

| | |
|---|---|
| Marticulation Fee (but once) | $ 5.00 |
| Professors' Tickets | 75.00 |
| Dissecting Ticket, including Material | 10.00 |
| Analytical Chemistry | 10.00 |
| Diploma Fee | 25.00 |
| Spring Course Ticket | 20.00 |

For further information and announcements, address

**H. A. SMITH, Dean, 128 Garfield Place, Cincinnati, Ohio.**

### DIRECTORY OF DENTAL SOCIETIES.

*American Dental Association*—Meets on the first Tuesday of August, 1890, at Excelsior Springs, Mo. Pres., M. W. Foster, Baltimore, Md.; Rec. Sec., Geo. H. Cushing, Chicago.

*Southern Dental Association*—Meets in Atlanta, Ga., on the third Tuesday in July, 1890. Prest., J. C. Story, Dallas, Texas; Secretary, M. C. Marshall, Little Rock, Ark.

### STATE DENTAL SOCIETIES.

*Alabama Dental Association*—Meets annually. Next meeting at Birmingham, on the second Tuesday of April, 1890. Pres. J. C. Wilkerson, Selma; Sec. R. Y. Jones, Florence.

*California State Dental Association*—Third Tuesday in July, 1890. Pres., T. N. Iglehart; Rec. Sec., W. A. Knowles, San Francisco; Cor. Sec., J. J. Birge. Next Meeting in San Francisco.

*Colorado State Dental Society*—Meets first Tuesday in June, 1890. Pres., J. W. Grannis; Sec., H. P. Kellog, Denver. Next meeting will be held in Denver

*Connecticut State Dental Society*—Pres. E. A. Stebbins, Shelburne Falls; Rec. Sec. Geo. A. Maxfield, Holyoke. Annual meeting Oct. 27th and 28th, 1889, at Hartford.

*Dental Society of the State of Maryland and District of Columbia*—Meets annually. Next meeting at Washington City, on the second Tuesday in October, 1889. Pres. B. F. Coy, of Baltimore: Sec. M. W. Foster, M. D.

*Georgia State Dental Society*—Meets second Tuesday in May, 1890, at Tybee; Pres S. A. White: Rec. Sec. H. H. Johnson; Cor. Sec. L. D. Carpenter, Atlanta.

*Illinois State Dental Society*—Meets at Springfield, second Tuesday in May, 1890. Pres., T. W. Pritchett, Chicago; Sec., G. Newkirk, Chicago.

*Indiana State Dental Society*-Meets next at Lake Maxintuckee on the last Tuesday of June, 1890. Pres., T. A. Goodwin, Warsaw, Ind.; Sec. Robt. W. Van Valzah, Terre Haute.

*Iowa State Dental Society*—Meets annually. Next meeting in Dubuque, on the first Tuesday in May, 1890. Pres. F. M. Shriver, Glenwood; Rec. Sec. G. W. Miller, Des Moines.

*Kentucky State Dental Association*—Meets annually, first Tuesday in June, 1890. Next meeting in Louisville. Pres., J. W. Wallace, Louisville; Sec., J. H. Baldwin, Louisville

*Kansas State Dental Association*—Pres. W. M. Shirley, Hiawatha; Sec. C. B. Gunn, Leavenworth. Next meeting will be held at Topeka, April 30th, 1890.

*Maine Dental Society*—Meets in August, at Thomaston. Pres. E. J. Robert, Augusta, Sec. C. E. Hussey, Beddeford.

*Maryland State Dental Association*—Meets annually in December. Pres. J. Emory Scott; Rec. Sec. W. W. Dunbracco; Cor, Sec.

*Massachusetts Dental Society*—Meets Semi-Annually in Boston, June 5th, 6th and 7th, 1890. Pres. G. A. Gerry, Boston; Rec. Sec. E. O. Kinsman, Boston.

*Michigan State Dental Association*—Meets annually. Next meeting at Jackson, Tuesday, June 3rd, 1890. Pres. C. S. Case, Jackson; Sec. Wm. Cleland, Detroit; Treas. H. K. Lathrop, Detroit.

*Mississippi State Dental Association*—Meets at Biloxi, 1890, during Camp Meeting. Pres., J. D. Miller, Vicksburg; Sec., A. H. Hilzim, Jackson.

*Minnesota State Society*—Pres. G. V. I. Brown, Duluth; Sec. C. H. Robinson, Wabash. Meets in Minneapolis, July 15th, 1890.

*Missouri Dental Association*—Meets at Pertel Springs, first Tuesday after July 4th, 1890. Pres., H. Fisher, St. Louis; Sec., John G. Harper, St. Louis.

*Nebraska State Dental Society*—Meets annually. Next meeting third Tuesday in May 1890, at Beatrice; Pres. J. J. Willey, Wahoo; Sec. C. R. Tefft, Lincoln.

*New Jersey State Dental Society*—Meets annually. Next meeting at Asbury Park, on the third Wednesday in July, 1890. Pres. S. C. G. Watkins, Mount Clair; Sec. C. A. Meeker, Newark.

*New Hampshire Dental Society*—Meets in Concord on the third Tuesday of June 1890. Pres., G. C. Duncan, East Jaffrey; Sec., E. B. Davis, Concord.

*North Carolina State Dental Society*—Meets in Wilmington, on the fourth Wednesday in June, 1890. Pres., S. P. Hilliard; Sec., H. C. Hearing, Concord.

*North Dakota Dental Society*—Meets ———, 1890. Pres., I. J. Hill, Fargo; Sec., J. W. Cloes, Jamestown.

*Ohio State Dental Society*—Meets annually. Next meeting at Columbus, last Tuesday of October, 1890. Pres. J. N. Sedgwick, Granville; Sec. J. R. Callahan, Cincinnati.

*P nnsylvania State Dental Society*—Meets second Tuesday of September, 1889, at Philadelphia; Pres. W. H. Roop Philadelphia; Rec. and Cor. Sec. Th. F. Chupein, Philadelphia.

*Rhode Island Dental Association*—Pres., H. W. Gillett; Sec., W. H. Carpenter. Meets quarterly, first Tuesdayin July, October, January, and April. Annual Meeting first Tuesday in January, at Providence.

*South Carolina State Dental Association*—Meets annually. Pres. L. S. Wolfe, Orangburg; Cor. Sec. E. C. Ridgell, Batesburg; Rec. Sec. R. A. Smith, Charleston. Next meeting at Greenville, third Tuesday in July, 1890.

*Texas State Dental Association*—Meets in Buton, first Tuesday in May, 1890. Pres., M. S. Read, Corsicana; Sec., C. B. Lewis, Dallas.

*Tennessee Dental Association*—Meets Annually. Next meeting at Columbia, first Tuesday in July 1890. Pres. W. H. Morgan, Nashville; Rec. Sec. A. G. White, Springfield; Cor. Sec. E. G. Grant, Columbia.

*The Dental Society of the State of New York*—Meets annually on the second Wednesday in May. Next session at Albany, May 8, 1890. Pres. J. Edw. Line, Rochester; Rec. Sec., F. T. Van Wert, Brooklyn; Cor. Sec., G. L. Curtis, Syracuse.

*Vermont State Dental Society*—Meets annually. Next meeting at Bellow's Falls, March 19, 1890. Pres. W. H. Spence, Poultney; Sec. Thos. Maund, Rutland.

*Virginia State Dental Association*—Meets in Charlottsville, Tuesday, August 19, 1890. Pres. J. Hall Moore; Sec. L. M. Cowarden, Richmond.

*Wisconsin State Dental Society*—Meets annually. Next meeting at Milwaukee, July 20, 1890. Pres. B. G. Marklein, Milwaukee; Sec. C. A. Southwell, Appleton, Wis.

### LOCAL SOCIETIES.

*American Academy of Dental Science*—Meets annually in Boston, Mass., on the first Wednesday in October, 1890. Pres. J. H. Batchelder; Rec. Sec. E. E. Hopkins; Cor. Sec. E. B. Hitchcock, Boston.

*Brooklyn Dental Society and Second District Society United*—Meets quarterly—Pres. T. H. Holly; Rec. Sec. A. N. Russell, Brooklyn.

*Central Dental Association of Northern New Jersey*—Meets bi-monthly; and annually in February. Pres. Oscar Adelburg, Elizabeth; Sec. Geo. Emory Adams, South Orange.

*Central Illinois Dental Society*—Meets annually. Next meeting at Lincoln, Oct. Oct. 9 and 10, 1889; Pres. J. M. Fishburn, El Paso; Sec. W. A. Johnston, Peoria.

*Chicago Dental Society*—Meets monthly. Pres. P. J. Kester; Sec. A. E. Baldwin

*Chicago Dental Club*—Meets on the fourth Monday of each month. Pres. A. B. Freeman; Sec. C. Stoddard Smith.

*Cleveland Dental Society.*—Meets the first Monday of each month. Pres. S. B. Dewey; Sec. Martha J. Robinson.

*Connecticut Valley Dental Society*—Meets 27 and 28 of Oct. 1889, at Savin Rock Conn. Pres. C. T. Stockwell, Springfield, Mass. Sec. A. M. Ross, Chicopee.

*Detroit Dental Society*—Meets monthly, Pres. E. C. Moore, Sec. Wm. Cleland.

*Eighth District Dental Society, N. Y.*—Meets semi-annually. Next meeting in Syracuse, Oct. 24-26, 1889. Pres. T. E. Howard; Sec. S. A. Freeman, Buffalo.

*East Tennessee Dental Association*—Meets annually. Next meeting will be held at Chattanooga, Tenn., on the first Tuesday of Aug., 1890. Pres. R. A. B. Noyers, Sec. S. N. Prothro.

*Mad River Dental Society*—Meets annually. Next meeting at Dayton, Tuesday, May 19, 1889. Pres. C. Bradley, Dayton; Sec. and Treas. L. C. Adams, Dayton,

*Mississippi Valley Dental Society*—Meets annually at Cincinnati, on first Wednesday in March, 1891. Pres. M. H. Fletcher, Cincinnati; Sec. H. T. Smith, Cincinnati.

*New England Dental Society*—Meets annually. Time and place of next meeting in Boston, November, 1890. Pres., C. W. Clement, Manchester, N. H.; Sec. E. O. Kinsman, Cambridge, Mass.

*Northern Illinois Dental Society* —Meets annually. Next meeting at Freeport, October 15th, 1890. Pres. G. W. Dennis, La Salle. Sec. T. W. Beckwith.

*Northern Ohio Dental Association*—Meets annually. Next meeting at Canton, O. on the second Tuesday in May, 1890. Pres. John Stephan, Cleveland, O.; Rec. Sec. F. F. Doud; Cor. Sec. S. B. Dewey, Cleveland, O.

*Northwestern Dental Association* (embraces portions of Dakota, Minnesota and Manitoba) meets annually. Next meeting at Detroit, Dak. on the fourth Tuesday in July, 1890. Pres. J. W. Cloes, Jamestown, Dak. Sec. S. J. Hill, Fargo, Dak.

*Odontological Society of Pennsylvania*—Meets on the first Saturday evening of each month, at 8 o'clock p. m., at 13th and Arch sts; Pres. Jas. Truman, Rec. Sec. A. W. Deane.

*Odontological Society of New York City*—Meets the third Tuesday of each month. Pres. Wm. Jarvie; Rec. Sec. E. T. Payne.

*Oaontological Society of Cincinnati*—Meets monthly. Pres. C. M, Wright; Sec W. M. Williams.

*Ohio Valley Dental Society.*—Meets quarterly. Next meeting first Monday in July, 1890, at Stuebenville, O. Pres. N. H. Harrison, Cadiz; Sec'y F. S. Maxwell, Stuebenville, O.

*Pennsylvania Association of Dental Surgeons*—Meets second Tuesday of each month; Pres. H. E. Roberts; Cor. Sec. and Reporter, W. N. Truman.

*Pittsburg Dental Society*—Meets the last Tuesday Evening of each month. Pres. J. B. Williams, Homestead; Sec. N. L. Reinecke.

*Fifth District Dental Society of N. Y.*—Meets semi-annually. Next meeting in Syracuse, October, 24-26. Pres. R. F. Jones, Utica; Sec. T. B. Mason, Phoenix.

*San Francisco Dental Society*—Meets the second Monday evening of each month. Pres. W. A. Knowles; Sec. Chas. Morffew; Treas. J. J. Birge.

*Sixth District Dental Society of N. Y.*—Meets semi-annually. Next meeting at Syracuse, Oct. 24-26, 1889. Pres. E. D. Downs, Owego; Sec. Myron D. Jewell, Richfield Springs.

*Southern Minnesota Dental Society*—Meets Semi-annualy in October and April. Next meeting in Waseca. Pres. C. H. Stearns, Zumbrota; Sec. H. L. Cruttenden, Northfield.

*St. Louis Dental Society*—Meets first and third Tuesday in each month. Pres. John G. Harper; Cor. Sec. Emma E. Chase; Rec. Sec. DeCoursey Lindsley.

*The Minneapolis Dental Society Monthly*—Pres. E. F. Clark, Sec. E. J. Morrison.

*Washington Dental Society*—Meets second Tuesday of each month. Pres. R. Finley Hunt; Rec. Sec. H. M. Schooly.

*First District Dental Society of the State of New York*—Meets annually. Pres. A. I. Northrop; Sec. J. E. Dexter, New York.

*Washington City Dental Society*—Pres. M. B. Noble; Sec. Wm. Donally. Meets monthly.

*The Western Illinois District Dental Association*—Meets at Kewanee, October 16, Pres. F. Christianer, Abington; Sec. R. W. Bailey, Macomb.

ESTABLISHED 1845.

### Ohio College of Dental Surgery.

Department of Dentistry—University of Cincinnati.

### SESSION 1888-89.

### FACULTY.

J. S. CASSIDY, M.D., D D.S., Professor of Chemistry and Materia Medica.
H. A. SMITH, D.D.S., DEAN OF THE FACULTY, Professor of Operative Dentistry and Dental Pathology.
C. M. WRIGHT, D.D.S., Professor of Physiology and General Pathology.
WM. KNIGHT, M.D., D.D.S., Professor of Anatomy and Oral Surgery.
GRANT MOLLYNEAUX, D.D.S., Professor of Mechanical Dentistry and Metallurgy.

### DEMONSTRATORS.

A. O. ROSS, D.D.S., } Demonstrators of Operative Dentistry.
F. L. CARY, D.D.S., }
G. S. JUNKERMAN, M.D., D.D.S., Demonstrator of Analytical Chemistry.
H. C. MATLACK, D.D.S., Demonstrator of Anatomy.
G. W. GANDEE, D.D.S., Demonstrator of Mechanical Dentistry.

### The Forty-Fourth Annual Session

BEGINS TUESDAY, OCT. 1st, 1889.

### FEES.

| | |
|---|---|
| Marticulation Fee (but once) | $ 5.00 |
| Professors' Tickets | 75.00 |
| Dissecting Ticket, including Material | 10.00 |
| Analytical Chemistry | 10.00 |
| Diploma Fee | 25.00 |
| Spring Course Ticket | 20.00 |

For further information and announcements, address

H. A. SMITH, Dean, 128 Garfield Place, Cincinnati, Ohio.

### DIRECTORY OF DENTAL SOCIETIES.

*American Dental Association*—Meets on the first Tuesday of August, 1891, place not selected. Pres., A. W. Harlan, Rec. Sec., Geo. H. Cushing, Chicago.

*Southern Dental Association*—Meets in Moorehead City, N. C., on the 7th of August, 1891. Prest., G. F. S. Wright, S. Carolina; Secretary, M. C. Marshall, Little Rock, Ark.

### STATE DENTAL SOCIETIES.

*Alabama Dental Association*—Meets annually. Next meeting at Anniston, on the second Tuesday of April, 1891. Pres. R. C. Young, Florence; Sec. J. H. Allen, Birmingham.

*California State Dental Association*—Third Tuesday in July, 1891. Pres., F. W. Bliss; Rec. Sec., W. A. Knowles, San Francisco; Cor. Sec., L. Van Orden. Next Meeting in San Francisco.

*Colorado State Dental Society*—Meets first Tuesday in June, 1891. Pres., J. W. Grannis; Sec., H. P. Kellog, Denver. Next meeting will be held in Denver

*Connecticut State Dental Society*—Pres. E. A. Stebbins, Shelburne Falls; Rec. Sec. Geo. A. Maxfield, Holyoke. Annual meeting Oct. 27th and 28th, 1891, at Hartford.

*Dental Society of the State of Maryland and District of Columbia*—Meets annually. Next meeting at Washington City, on the second Tuesday in October, 1891. Pres. B. F. Coy, of Baltimore: Sec. M. W. Foster, M. D.

*Georgia State Dental Society*—Meets second Tuesday in July, 1891, at Gainsville; Pres. S. B. Garfield: Rec. Sec. C. A. Ryder; Cor. Sec. L. D. Carpenter. Atlanta.

*Illinois State Dental Society*—Meets at Springfield, second Tuesday in May, 1891. Pres., T. W. Pritchett, Chicago; Sec., G. Newkirk, Chicago.

*Indiana State Dental Society*—Meets next at Indianapolis, on the last Tuesday of June, 1891. Pres., C. A. Budd, Muncie, Ind.; Sec. Robt. W. Van Valzah, Terre Haute.

*Iowa State Dental Society*—Meets annually. Next meeting in Sioux City, on the first Tuesday in May, 1891. Pres. E. J. Peterson, Dubuque; Rec. Sec. S. B. Hatch, Sioux City

*Kentucky State Dental Association*—Meets annually, first Tuesday in June, 1891. Next meeting in Louisville. Pres., J. W. Wallace, Louisville; Sec., J. H. Baldwin, Louisville

*Kansas State Dental Association*—Pres. W. M. Shirley, Hiawatha; Sec. C. B. Gunn, Leavenworth. Next meeting will be held at Topeka, April 30th, 1891.

*Maine Dental Society*—Pres. E. Bacon, Portland, Sec. C. E. Bryant, Pittsfield. Next meeting in Brunswick, on the third Tuesday in July, 1891.

*Maryland State Dental Association*—Meets annually in December. Pres. J. Emory Scott; Rec. Sec. W. W. Dunbracco; Cor. Sec.

*Massachusetts Dental Society*—Meets Semi-Annually in Boston, June 5th, 6th and 7th, 1891. Pres. G. A. Gerry, Boston; Rec. Sec. E. O. Kinsman, Boston.

*Michigan State Dental Association*—Meets annually. Next meeting at Jackson, Tuesday, June 3rd, 1891. Pres., H. K. Lathrop: Detroit; Sec., J. Ward House, Grand Rapids. Treas. Geo. H. Mosher, Jackson.

*Mississippi State Dental Association*—Meets July 18th, at Biloxi, 1891, during Camp Meeting. Pres., R. K. Luckie, Holly Springs; Sec., R. H. Hight, Grenada.

*Minnesota State Society*—Pres. M. G. Jenison, Minneapolis; Sec. L. D. Leonard, Minneapolis. Meets in Minneapolis, July 9th, 1891.

*Missouri Dental Association* Meets at Louisiana, Mo., first Tuesday after July 7th, 1891, Pres., J. F. McWilliams, Mexico; Sec., John G. Harper, St. Louis.

*Nebraska State Dental Society*—Meets annually. Next meeting third Tuesday in May 1891, at Beatrice; Pres. J. J. Willey, Wahoo; Sec. C. R. Tefft, Lincoln.

*New Jersey State Dental Society*—Meets annually. Next meeting at Asbury Park on the third Wednesday in July, 1891. Pres. G. E. Adams, S. Orange; Sec. C. A. Meeker, Newark.

*New Hampshire Dental Society*—Meets—Time to be decided. Pres., W. R. Blackstone, Manchester; Sec., E. B. Davis, Concord.

*North Carolina State Dental Society*—Meets in Durham, first Tuesday in May, 1891. Pres., H. C. Hearing, Concord; Sec., C. A. Rominger, Hillsboro.

*North Dakota Dental Society*—Meets ———, 1891. Pres., I. J. Hill, Fargo; Sec., J. W. Cloes, Jamestown.

*Ohio State Dental Society*—Meets annually. Next meeting at Columbus, last Tuesday of October, 1891. Pres. E. G. Betty, Cincinnati; Sec. Otto Arnold, Columbus.

*Pennsylvania State Dental Society*—Meets second Tuesday of September, 1891, at Philadelphia; Pres. W. H. Roop Philadelphia; Rec. and Cor. Sec. Th. F. Chupein, Philadelphia.

*Rhode Island Dental Association*—Pres., D. F. Keefe, Providence; Sec., W. H. Carpenter, Providence. Meets quarterly, second Tuesday in July, October, January and April. Annual Meeting second Tuesday in July, at Providence.

*South Carolina State Dental Association*—Meets annually. Pres. L. S. Wolfe, Orangburg; Cor. Sec. E. C. Ridgell, Batesburg; Rec. Sec. R. A. Smith, Charleston. Next meeting at Greenville, third Tuesday in July, 1891.

*Texas State Dental Association*—Meets in Waco, fourth Tuesday in May, 1891. Pres., J. H. Laseter,; Sec., C. B. Lewis, Dallas.

*Tennessee Dental Association*—Meets Annually. Next meeting at Columbia, first Tuesday in July 1891. Pres. W. H. Morgan, Nashville; Rec. Sec. A. G. White, Springfield; Cor. Sec. E. G. Grant, Columbia.

*The Dental Society of the State of New York*—Meets annually on the second Wednesday in May. Next session at Albany, May 8, 1891. Pres. W. W. Walker, New York; Rec. Sec., F. T. Van Woert, Brooklyn; Cor. Sec., R. Ottolengui, New York.

*Vermont State Dental Society*—Meets annually. Next meeting at Rutland, March 18, 1891. Pres. G. W. Hoffman, White River Jct.; Sec. Thos. Mound, Rutland.

*Virginia State Dental Association*—Meets in Charlottsville, Tuesday, August 19, 1891. Pres. J. Hall Moore; Sec. L. M. Cowarden, Richmond.

*Wisconsin State Dental Society*—Meets annually. Next meeting at Milwaukee, July 20, 1891. Pres. B. G. Marklein, Milwaukee; Sec. C. A. Southwell, Appleton, Wis.

### LOCAL SOCIETIES.

*American Academy of Dental Science*—Meets annually in Boston, Mass., on the first Wednesday in October, 1890. Pres. J. H. Batchelder; Rec. Sec. E. E. Hopkins; Cor. Sec. E. B. Hitchcock, Boston.

*Brooklyn Dental Society and Second District Society United*—Meets quarterly—Pres. T. H. Holly; Rec. Sec. A. N. Russell, Brooklyn.

*Central Dental Association of Northern New Jersey*—Meets bi-monthly; and annually in February. Pres. Oscar Adelburg, Elizabeth; Sec. Geo. Emory Adams, South Orange.

*Central Illinois Dental Society*—Meets annually. Next meeting at Lincoln, Oct. Oct. 9 and 10, 1891; Pres. J. M. Fishburn, El Paso; Sec. W. A. Johnston, Peoria.

*Chicago Dental Society*—Meets monthly. Pres. C. N. Johnson; Sec. A. E. Baldwin

*Chicago Dental Club*—Meets on the fourth Monday of each month. Pres. A. B. Freeman; Sec. C. Stoddard Smith.

*Cleveland Dental Society.*—Meets the first Monday of each month. Pres. S. B. Dewey; Sec. Martha J. Robinson.

*Connecticut Valley Dental Society*—Meets 27 and 28 of Oct. 1891, at Savin Rock Conn. Pres. C. T. Stockwell, Springfield, Mass. Sec. A. M. Ross, Chicopee.

*Detroit Dental Society*—Meets monthly, Pres. E. C. Moore, Sec. Wm. Cleland.

*Eighth District Dental Society, N. Y.*—Annual meeting third Tuesday in April, 1891, Union Dental Convention, Oct. 28, 1891 in Rochester. Pres. C. S. Butler, Buffalo, Sec. W. A. Barrows, Buffalo.

*East Tennessee Dental Association*—Meets annually. Next meeting will be held at Chattanooga, Tenn., on the first Tuesday of Aug., 1891. Pres. R. A. B. Noyers, Sec. S. N. Prothro.

*Mississippi Valley Dental Society*—Meets annually at Cincinnati, on first Wednesday in March, 1891. Pres. M. H. Fletcher, Cincinnati; Sec. H. T. Smith, Cincinnati.

*New England Dental Society*—Meets annually. Time and place of next meeting in Boston, November, 1890. Pres., C. W. Clement, Manchester, N. H.; Sec. E. O. Kinsman, Cambridge, Mass.

*Northern Illinois Dental Society*.—Meets annually. Next meeting at Freeport, October 15th, 1890. Pres. G. W. Dennis, La Salle. Sec. T. W. Beckwith.

*Northern Ohio Dental Association*—Meets annually. Next meeting at Oberlin, O. on the second Tuesday in May, 1891. Pres. F. S. Whitslar, Youngstown, O.; Rec. Sec. F. F. Doud; Cor. Sec. R. H. Barnes, Cleveland, O.

*Northwestern Dental Association* (embraces portions of Dakota, Minnesota and Manitoba) meets annually. Next meeting at Detroit, Dak. on the fourth Tuesday in July, 1891. Pres. J. W. Cloes, Jamestown, Dak. Sec. S. J. Hill, Fargo, Dak.

*Odontological Society of Pennsylvania*—Meets on the first Saturday evening of each month, at 8 o'clock p. m., at 13th and Arch sts; Pres. Jas. Truman, Rec. Sec. A. W. Deane.

*Odontological Society of New York City*—Meets the third Tuesday of each month. Pres. Wm. Jarvie; Rec. Sec. E. T. Payne.

*Odontological Society of Cincinnati*—Meets monthly. Pres. C. M. Wright; Sec. W. M. Williams.

*Odontological Society of Chicago*—Meets monthly. Pres. J. Kester; Sec. E. Noyes.

*Ohio Valley Dental Society*.—Meets quarterly. Next meeting first Monday in July, 1891, at Stuebenville, O. Pres. N. H. Harrison, Cadiz; Sec'y F. S. Maxwell, Stuebenville, O.

*Pennsylvania Association of Dental Surgeons*—Meets second Tuesday of each month; Pres. H. E. Roberts; Cor. Sec. Theo. F. Chupein.

*Pittsburg Dental Society*—Meets the last Tuesday Evening of each month. Pres. J. B. Williams, Homestead; Sec. N. L. Reinecke.

*Fifth District Dental Society of N. Y.*—Meets semi-annually. Next meeting in Syracuse, October, 24-26. Pres. R. F. Jones, Utica; Sec. T. B. Mason, Phoenix.

*San Francisco Dental Society*—Meets the second Monday evening of each month. Pres. W. A. Knowles; Sec. Chas. Morffew; Treas. J. J. Birge.

*Sixth District Dental Society of N. Y.*—Meets semi-annually. Next meeting at Syracuse, Oct. 24-26, 1889. Pres. Wm. H. Hall, Binghamton; Sec. Myron D. Jewell, Richfield Springs.

*Southern Minnesota Dental Society*—Next meeting in Mankato, third Tuesday in April, 1891. Pres. C. W. Nutting, Spring Valley; Sec. C. L. Harris, Mankato.

*St. Louis Dental Society*—Meets first and third Tuesday in each month. Pres. John G. Harper; Cor. Sec. Emma E. Chase; Rec. Sec. DeCoursey Lindsley.

*The Minneapolis Dental Society Monthly*—Pres. E. F. Clark, Sec. E. J. Morrison.

*Washington Dental Society*—Meets second Tuesday of each month. Pres. R. Finley Hunt; Rec. Sec. H. M. Schooly.

*First District Dental Society of the State of New York*—Meets annually. Pres. A. L. Northrop; Sec. J. E. Dexter, New York.

*Washington City Dental Society*—Pres. M. B. Noble; Sec. Wm. Donally. Meets monthly.

*The Western Illinois District Dental Association*—Meets at Kewanee, October 16. Pres. F. Christianer, Abington; Sec. R. W. Bailey, Macomb.

### CONTENTS.

### COMMUNICATIONS.

### THE DENTAL REGISTER,

A MONTHLY MAGAZINE DEVOTED TO THE INTERESTS OF THE

### DENTAL PROFESSION.

*Terms Reduced to* $2.00 *Per Year. Single Copies,* 25 *Cents.*

**EDITED BY PROF. J. TAFT, D.D.S.**

—AND—

**Published by SAM'L A. CROCKER & CO., Ohio Dental and Surgical Depot.**

The volume commences in January and closes in December.

The REGISTER being issued monthly is always fresh, therefore Indispensable to the practitioner who desires to keep posted up with the new inventions and improvements which are daily developed. Neither expense nor effort will be spared to give value and variety to its contents. Articles will be illustrated with well executed engravings whenever they will add to the interest or assist to explanation.

Its extensive circulation and frequency of issue make it one of the BEST DENTAL ADVERTISING MEDIUMS in the United States.

All subscriptions must begin with January or July.

Local or special notices, five lines or less, will be inserted for $3 each insertion; over five lines, 50 cts. per line.

All advertising bills payable in advance, or at furthest, after the issue of the first number containing the advertisement. All transient advertisements to be paid for in advance.

☞CONTRIBUTIONS SOLICITED.

Exclusively Editorial Communications should be addressed to the Editor.

The latest number of the REGISTER may be ordered with or without other goods at any time, and all letters should be addressed to

SAM'L A. CROCKER & CO., Ohio Dental and Surgical Depot, Cincinnati, O.

